# Ascorbate oxidation by iron, copper and reactive oxygen species: review, model development, and derivation of key rate constants

**DOI:** 10.1038/s41598-021-86477-8

**Published:** 2021-04-01

**Authors:** Jiaqi Shen, Paul T. Griffiths, Steven J. Campbell, Battist Utinger, Markus Kalberer, Suzanne E. Paulson

**Affiliations:** 1 Department of Atmospheric and Oceanic Sciences, University of California At Los Angeles, Los Angeles, CA 90095-1565 USA; 2 Department of Chemistry, Cambridge University, Lensfield Rd, Cambridge, CB2 1EW UK; 3 Department of Environmental Sciences, University of Basel, Klingelbergstrasse 27, 4056 Basel, Switzerland

**Keywords:** Environmental sciences, Chemistry

## Abstract

Ascorbic acid is among the most abundant antioxidants in the lung, where it likely plays a key role in the mechanism by which particulate air pollution initiates a biological response. Because ascorbic acid is a highly redox active species, it engages in a far more complex web of reactions than a typical organic molecule, reacting with oxidants such as the hydroxyl radical as well as redox-active transition metals such as iron and copper. The literature provides a solid outline for this chemistry, but there are large disagreements about mechanisms, stoichiometries and reaction rates, particularly for the transition metal reactions. Here we synthesize the literature, develop a chemical kinetics model, and use seven sets of laboratory measurements to constrain mechanisms for the iron and copper reactions and derive key rate constants. We find that micromolar concentrations of iron(III) and copper(II) are more important sinks for ascorbic acid (both AH_2_ and AH^−^) than reactive oxygen species. The iron and copper reactions are catalytic rather than redox reactions, and have unit stoichiometries: Fe(III)/Cu(II) + AH_2_/AH^−^  + O_2_ → Fe(III)/Cu(II) + H_2_O_2_ + products. Rate constants are 5.7 × 10^4^ and 4.7 × 10^4^ M^−2^ s^−1^ for Fe(III) + AH_2_/AH^−^ and 7.7 × 10^4^ and 2.8 × 10^6^ M^−2^ s^−1^ for Cu(II) + AH_2_/AH^−^, respectively.

## Introduction

Ascorbic acid is of great interest in food, where it is both an essential vitamin and a natural preservative. Ascorbic acid is also vital for plants. It not only plays a role in photosynthesis, cell growth and signal transduction, but also helps defend from oxidative stress as the most abundant water-soluble antioxidant in plants^1–4^. Because of its importance for food and in plants, food chemists and botanists have performed the vast majority of studies of ascorbic acid oxidation chemistry^1–7^.

In mammalian systems, ascorbic acid is a common and important molecule with roles in metabolic function, oxidative stress responses and immune system maintenance taking place in epithelial lung lining fluid and other areas in the body^8, 9^.

In an air pollution context, inhaled particulate matter, a highly complex and variable mixture of inorganic and organic compounds, encounters the lung lining fluid containing substantial concentrations of ascorbic acid. Growing evidence indicates that transition metals in inhaled particles are particularly active components capable of inducing a wide range of negative health effects including myocardial infarction, adverse birth outcomes and respiratory illnesses^10–12^. A leading hypothesis for how airborne particles induce health effects is via oxidative stress, and redox-active transition metals such as iron and copper have been heavily implicated in the ability of particles to generate reactive oxygen species and therefore potentially contribute to aerosol toxicity^13, 14^. For example, soluble iron and copper in synthetic lung fluid correlate with the formation of reactive oxygen species OH^⋅^ and H_2_O_2_^15, 16^.

Because ascorbic acid is a key antioxidant in lung lining fluid, ascorbic acid consumption is one of the assays used by atmospheric chemists to quantify aerosol oxidative potential^17^; aerosol oxidative potential is proving to be better at predicting adverse health outcomes than particle mass^18^. Ascorbate/ ascorbic acid consumption has been observed to be positively correlated with total iron and copper concentrations in ambient aerosol^14^.

Ascorbic acid can have both pro- and anti-oxidant roles, and it reacts with reactive oxygen species (ROS) and transition metals. Ascorbic acid can be readily oxidized by undergoing a one- or two-electron transfer, terminating the free radical-mediated chain reactions in foods and tissue, reducing lipid peroxidation and deterioration of foods^6^. The autoxidation of ascorbic acid by oxygen in the presence of transition metals, especially cupric (Cu(II)) and ferric (Fe(III)) ions accounts for the majority of loss of this ascorbic acid activity in food. Despite its role as an efficient antioxidant, ascorbic acid can also accelerate oxidative deterioration of flavor and color in food through Fenton-type radical reactions^6, 19^. This pro-oxidant effect occurs when transition metal ions are present, and the level of available ascorbic acid is relatively low and not sufficient to scavenge the radicals formed by Fenton-type reactions. In both food and physiological conditions, the key loss pathways for ascorbic acid are via ROS and transition metals, especially Fe(III) and Cu(II). However, for the transition metal reactions, the stoichiometries, mechanisms and rate constants are all very uncertain. Further, while the ROS ascorbic acid reactions are reasonably well understood from a mechanistic standpoint, the range of rate constants in the literature for these reactions spans about a factor of 15^20–25^.

Here we develop a model in the Kinetics Preprocessor (KPP)^26^ environment based on available ascorbic acid chemistry with ROS and free iron and copper chemistry from the literature. The model is validated against measurements of the formation of dehydroascorbic acid (DHA), the main oxidation product of ascorbic acid in the presence of micromolar concentrations of Fe(II), Fe(III) and Cu(II) at pH 2.8 and 7.0. The measurements at pH 2.8 were made to develop an online measurement of ascorbic acid consumption by ambient particulate matter^17^, and allow us to probe the chemistry of ascorbic acid, AH_2_. Additional measurements were made at pH 7.0 to probe the reactions of the deprotonated form, AH^−^. We also use measurements of ascorbate loss and/or OH^**⋅**^ formation from Lin and Yu^27^ and Charrier and Anastasio^15^ at ~ pH 7 to further constrain the model. We then use the model to constrain the mechanisms and derive rate constants for the catalytic reactions of Fe(III) and Cu(II) with both AH_2_ and AH^−^ in the presence of oxygen.

## Ascorbic acid chemistry review

Here we use ‘ascorbic acid’ to mean the sum of the protonated form, AH_2_ and the deprotonated form AH^−^, and the chemical formulas to indicate the individual species.

### pH dependence

**Figure 1:**
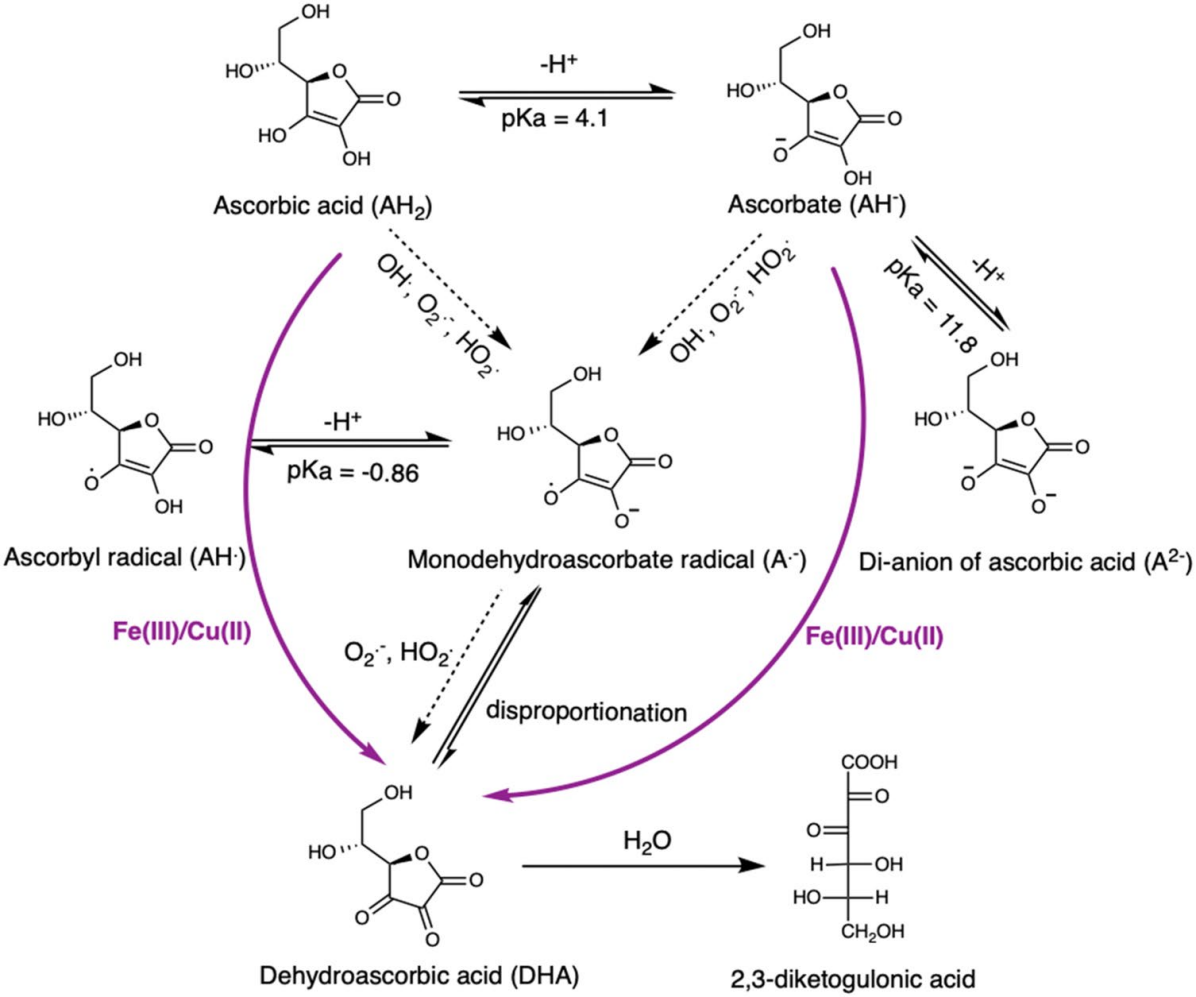
Ascorbic acid oxidation scheme.

Ascorbic acid reacts with several species of ROS, as well as the oxidized forms of several transition metals (Fig. 1). As ascorbic acid (AH_2_) can readily lose a proton to form the ascorbate anion (AH^−^), (pK_a,1_ = 4.1; pK_a,2_ = 11.8) both AH_2_ and AH^−^ play roles in chemistry at low and neutral pHs. Typically, AH_2_ and AH^−^ reactions with ROS and transition metals have rate constants that differ by up to several orders of magnitude; with the reactions of AH^−^ usually being faster. Additionally, the pK_a_ of HO_2_^**⋅**^ is 4.8 (R36, Table 2); the reaction rates for HO_2_^**⋅**^ and O_2_^**⋅**−^ also differ by up to three orders of magnitude. As a result of these effects and others, the ascorbic acid oxidation reactions are fairly sensitive to pH.

### Reactions with hydroxyl and hydroperoxyl radicals and superoxide

OH^**⋅**^ reactions with both AH_2_ and AH^−^ (Table 1, R4 and R7) appear to proceed at close to diffusion-controlled collision rates. The rate constants for these reactions fall in the ranges (4.5–7.9) × 10^9^ M^−1^ s^−1^ at pH 1–1.5 and (1–11) × 10^9^ M^−1^ s^−1^ at pH 7–11 respectively (Supplementary Table S1). We adopt the rate constant of 7.9 × 10^9^ M^−1^ s^−1^ from Redpath and Willson^21^ for the oxidation of AH_2_ by OH^**⋅**^ and 1.1 × 10^10^ M^−1^ s^−1^ from Buettner and Schafer^22^ for AH^−^ in our study, because the deprotonated ascorbic acid tends to react more rapidly than the protonated form.

**Table 1:** Ascorbic acid model.

#	Reaction	$k_{f}$	$k_{r}$	$K_{eq}$
**Ascorbate chemistry**				
1a	${\text{AH}}_{2} \rightleftharpoons {\text{AH}}^{ - } + {\text{H}}^{ + }$			7.94E−5
2a	${\text{AH}}^{ - } \rightleftharpoons {\text{A}}^{2 - } + {\text{H}}^{ + }$			1.58E−12
3a	${\text{AH}}^{ \cdot } \rightleftharpoons {\text{A}}^{ \cdot - } + {\text{H}}^{ + }$			7.24
4b	${\text{AH}}_{2} + {\text{OH}}^{ \cdot } = {\text{A}}^{ \cdot - } + {\text{H}}_{2} {\text{O}} + {\text{H}}^{ + }$	7.9E9		
5c	${\text{AH}}_{2} + {\text{HO}}_{2}^{.} = {\text{A}}^{ \cdot - } + {\text{H}}_{2} {\text{O}}_{2} + {\text{H}}^{ + }$	1.6E4		
6c	${\text{AH}}_{2} + {\text{O}}_{2}^{ \cdot - } = {\text{A}}^{ \cdot - } + {\text{H}}_{2} {\text{O}}_{2}$	0.2k_6_ + k_8_ = 1.22E7		
7a	${\text{AH}}^{ - } + {\text{OH}}^{ \cdot } = {\text{A}}^{ \cdot - } + {\text{H}}_{2} {\text{O}}$	1.1E10		
8c	${\text{AH}}^{ - } + {\text{ HO}}_{2}^{ \cdot } = {\text{A}}^{ \cdot - } + {\text{H}}_{2} {\text{O}}_{2}$			
9c	${\text{AH}}^{ - } + {\text{ O}}_{2}^{ \cdot - } = {\text{A}}^{ \cdot - } + {\text{HO}}_{2}^{ - }$	5E4		
10c	${\text{A}}^{ \cdot - } + {\text{HO}}_{2}^{.} = {\text{DHA}} + {\text{ HO}}_{2}^{ - }$	5E9		
11c	${\text{A}}^{ \cdot - } + {\text{O}}_{2}^{ \cdot - } = {\text{ DHA}} + {\text{HO}}_{2}^{ - } - {\text{H}}^{ + }$	2.6E8		
12d,e	$2{\text{A}}^{ \cdot - } \rightleftharpoons {\text{AH}}^{ - } + {\text{DHA}} - {\text{H}}^{ + }$	7E4	4.2E−12	1.67E16
13d,e	$2{\text{A}}^{ \cdot - } \rightleftharpoons {\text{AH}}_{2} + {\text{DHA}} - 2{\text{H}}^{ + }$	8E7	3.8E−13	2.1E20
14	$${\text{Fe}}\left( {{\text{III}}} \right)^{{**}} + {\text{AH}}_{2} + {\text{O}}_{2} = {\text{Fe}}\left( {{\text{III}}} \right)^{{**}} + {\text{DHA}} + {\text{H}}_{2} {\text{O}}_{2}$$	5.7E4 This study		
15	$${\text{Fe}}\left( {{\text{III}}} \right)^{{**}} + {\text{AH}}^{ - } + {\text{O}}_{2} = {\text{Fe}}\left( {{\text{III}}} \right)^{{**}} + {\text{DHA}} + {\text{H}}_{2} {\text{O}}_{2} - {\text{H}}^{ + }$$	4.7E4 This study		
16	$${\text{Cu}}\left( {{\text{II}}} \right)^{{**}} + {\text{ AH}}_{2} { } + {\text{O}}_{2} = {\text{Cu}}\left( {{\text{II}}} \right)^{{**}} + {\text{DHA}} + {\text{H}}_{2} {\text{O}}_{2}$$	7.7E4 This study		
17	$${\text{Cu}}\left( {{\text{II}}} \right)^{{**}} + {\text{AH}}^{ - } + {\text{O}}_{2} = {\text{Cu}}\left( {{\text{II}}} \right)^{{**}} + {\text{DHA}} + {\text{H}}_{2} {\text{O}}_{2} - {\text{H}}^{ + }$$	2.8E6 This study		
**DHA measurement and degradation reactions**				
18f	${\text{oPDA}} + {\text{DHA}} = {\text{DHA}} - {\text{oPDA}}$	4.6		
19z	${\text{DHA}} + {\text{ OH}}^{ \cdot } = {\text{product }}$	1E10		
20g	${\text{DHA}} + {\text{H}}_{2} {\text{O}} = {\text{DKG}}$	5.8E−4 at neutral pH		
21z	${\text{DKG}} + {\text{ OH}}^{ \cdot } = {\text{product}}$	1E10		
22h	${\text{DHA}} + {\text{H}}_{2} {\text{O}}_{2} = {\text{products}}$	4.2E−2		

Fe(III)** represents free Fe(III) species (${\text{Fe}}^{3 + }$, ${\text{FeOH}}^{2 + }$, ${\text{Fe}}\left( {{\text{OH}}} \right)_{2}^{ + }$, ${\text{FeCl}}^{2 + }$, ${\text{FeSO}}_{4}^{ + }$ and ${\text{Fe}}({\text{SO}}_{4} )_{2}^{ - }$).

Cu(II)** represents ${\text{Cu}}^{2 + }$, ${\text{CuOH}}^{ + }$, ${\text{CuSO}}_{4}$, ${\text{CuCl}}^{ + }$ and ${\text{CuCl}}_{2}$.

These forward and back reactions are written separately in the KPP input file.

^a^Buettner and Schafer^22^, ^b^Redpath and Willson^21^, ^c^Cabelli and Bielski^24^, ^d^Van der Zee and Van den Broek^36^, ^e^Bielski et al.^37^, ^f^Vislisel et al.^57^, ^g^Dewhirst and Fry^47^, ^h^Parsons et al.^45^, ^z^Estimate numbers.

Both AH_2_ and AH^−^ readily undergo one-electron oxidation by superoxide (O_2_^**.**−^), hydroperoxyl radical (HO_2_^**⋅**^) and hydroxyl radical (OH^**⋅**^) to form the ascorbate radical (A^**.**−^) (R4–9, Table 1). The pK_a_ of AH^.^ is sufficiently low that the protonated radical (AH^**.**^) can be ignored. The unpaired electron of A^**.**−^ residing in the π-system makes A^**.**−^ relatively unreactive^22^, however A^**.**−^ can form DHA via disproportionation (R12,13, Table 1).

The reactions of AH_2_ with HO_2_^**⋅**^ (Table 1 R5) and O_2_^**⋅**−^ (R6), and AH^−^ with HO_2_^**⋅**^ (R8) and O_2_^**⋅**−^ (R9) have been investigated by Nadezhdin and Dunford^25^ and Cabelli and Bielski^24^. The experimental data disagree by a factor of 1.5–15, although they have the same shape, with a maximum in the observed rate at about pH 4.5. Because both AH_2_ and the hydroperoxyl radical have similar pK_a_s (4.1 and 4.8 respectively), in the pH range 2–7, the contributions of R6 and R8 are difficult to separate, while at low and high pH R5 and R9 dominate, respectively. Because Nadezhdin and Dunford^25^ neglected the AH_2_ reactions (R5 and R6) in their discussion and their data span a smaller pH range, we use values based on the data and analysis by Cabelli and Bielski^24^. Cabelli and Bielski^24^ conclude that it is not possible to deconvolute k_6_ and k_8_, but the sum (0.356 k_6_ + k_8_) can be said to be equal to 1.22 × 10^7^ M^−1^ s^−1^. Because we use updated pK_a_s for ascorbic acid and HO_2_^**⋅**^ (Tables 1 and 2) we adjust this sum to be (0.200 k_6_ + k_8_) = 1.22 × 10^7^ M^−1^ s^−1^ for our model. As the transition metal systems we examined are not very sensitive to these values, we have not tested them further.

The ascorbate radical (A^**⋅**−^) can be further oxidized by HO_2_^**⋅**^ and O_2_^**⋅**−^ (R10 and R11, Table 1). The rate constants for reactions of A^**⋅**−^ and HO_2_^**⋅**^ and O_2_^**⋅**−^ were measured by Cabelli and Bielski^24^ using radiolysis; using pH to select HO_2_^**⋅**^ (pH = 1–3) or O_2_^**⋅**−^ (pH = 7.8–8), and were determined to be 5.0 × 10^9^ M^−1^ s^−1^ and 2.6 × 10^8^ M^−1^ s^−1^, respectively.

### Autooxidation

Ascorbic acid is an excellent electron-donor antioxidant. The relatively low reduction potential of ascorbate (0.19 V for DHA/AH^−^ at pH 3.5) should allow it to be readily oxidized by molecular oxygen^5^. However, while this redox reaction is thermodynamically favorable, it is spin forbidden; molecular oxygen is a triplet with two unpaired electrons, while ascorbate is in the ground state^5^. The only ascorbate species that is capable of true autoxidation, determined after treating the solutions with Chelex resin to remove trace metals appears to be the ascorbate dianion (A^2−^) + O_2_^9^. Because there is little A^2−^ at pHs below ~ 10 (Table 1 R2), the autoxidation rate for ascorbate (all forms) is slow, ~ 6 × 10^–7^ s^−1^ at pH 7^5^. We verified this as part of our measurements (not shown).

### Transition metal reactions

#### Catalytic or redox?

Ascorbate reactions with iron and copper are central to metal-mediated antioxidant chemistry, and it is clear that ascorbate reacts overwhelmingly with the oxidized forms of the metals (Fe(III) and Cu(II)). The early studies of this reaction uniformly interpreted their data with catalytic mechanisms^28–31^ such as:

1 $${\text{AH}}_{{2}} /{\text{AH}}^{ - } + {\text{ Fe}}\left( {{\text{III}}} \right)/{\text{Cu}}\left( {{\text{II}}} \right) \, + {\text{ O}}_{{2}} \to {\text{DHA }} + {\text{ Fe}}\left( {{\text{III}}} \right)/{\text{Cu}}\left( {{\text{II}}} \right) \, + {\text{H}}_{{2}} {\text{O}}_{{2}} \left( { - {\text{H}}^{ + } } \right).$$

Following this, Buettner^32^ reported rate constants for the bimolecular reactions of Fe(III) and Fe(III)/EDTA and Cu(II) with AH^−^ at pH 7 in oxygenated solution, and suggested it was a catalytic reaction, although the rate constants they reported did not include an oxygen dependence. Later, Buettner and Jurkiewicz^19^ instead described it as a redox reaction:

2 $${\text{AH}}_{{2}} /{\text{AH}}^{ - } + {\text{ Fe}}\left( {{\text{III}}} \right)/{\text{Cu}}\left( {{\text{II}}} \right) \to {\text{A}}^{ \cdot - } + {\text{ Fe}}\left( {{\text{II}}} \right)/{\text{Cu}}\left( {\text{I}} \right) \, + \, \left( {2} \right){\text{ H}}^{ + }$$

and suggested a somewhat higher rate constant for the Fe(III)/EDTA complex. Subsequent modeling studies adopted the redox reaction^33, 34^.

Overall, however, the ascorbate mechanistic literature does not support a significant role for the redox reaction. Most or all studies point to the catalytic reaction instead; this includes the original source of the rate constant used for the redox reaction in Buettner^32^, and the mechanistic studies described below. We also test the redox and catalytic mechanisms with our model (“Ascorbate oxidation via the catalytic, redox or OH^**⋅**^/HO_2_^**⋅**^/O_2_^**⋅**−^ Pathways”).

#### Transition metal ascorbic acid reaction mechanism

Three detailed mechanisms have been proposed to describe the oxidation process of ascorbic acid by iron and copper^28–31^. All of them begin with the oxidized form (Fe(III) or Cu(II)), consume oxygen, and produce A^**.**−^ or DHA plus a reduced form of oxygen (HO_2_^**.**^, O_2_^**.**−^ or H_2_O_2_). In the proposed mechanisms, metal, ascorbic acid and oxygen molecules form a complex, with the metal ion serving as a bridge that transfers one or two electrons from ascorbic acid to oxygen and maintains its valence. In Scheme A, proposed by Khan and Martell^31^, a ternary metal-ascorbate-oxygen complex forms in which one electron is transferred from AH_2_ or AH^−^ through metal ion to oxygen (Scheme 1).

##### Scheme A Khan and Martell^31^

3 $${\text{AH}}^{ - } + {\text{M}}^{{{\text{n}} + }} \rightleftharpoons \left[ {{\text{MAH}}} \right]^{{\left( {{\text{n}} - 1} \right) + }}$$

4 $$[{\text{MAH}}]^{{\left( {{\text{n}} - 1} \right) + }} + {\text{ O}}_{2} \rightleftharpoons \left[ {{\text{MAH}}\left( {{\text{O}}_{2} } \right)} \right]^{{\left( {{\text{n}} - 1} \right) + }}$$

5 $$\left[ {{\text{MAH}}\left( {{\text{O}}_{2} } \right)} \right]^{{\left( {{\text{n}} - 1} \right) + }} \to \left[ {{\text{M}}\dot{{\text{A}}}{\text{H}}\left( {\dot{{\text{O}}}_{2} } \right)} \right]^{{\left( {{\text{n}} - 1} \right) + }}$$

6 $$\left[ {{{\text{M}}}\dot{{\text{A}}}{{\text{H}}}\left( {\dot{{\text{O}}}_{2} } \right)} \right]^{{\left( {{{\text{n}}} - 1} \right) + }} \to {{\text{A}}}^{{. - }} + {{\text{ M}}}^{{{{\text{n}}} + }} + {{\text{HO}}}_{2}^{ \cdot }$$

Subsequently, Jameson and Blackburn^28^ and Jameson and Blackburn^29^ suggested a mechanism that involves an initial two-electron transfer to oxygen and the formation of Cu(III) intermediates (Supplementary Scheme B, Supplementary Eqs. (7)–(14)). Shtamm et al.^30^ then proposed a two-electron transfer mechanism involving Cu(I)–Cu(II) redox couple (Supplementary Scheme C). Although there is no agreement on the step by step oxidation state of metal ions in the catalytic cycle, there is some evidence showing that the reducibility of metal ion was necessary for it to be an active catalyst. Khan and Martell^31^ tested VO^2+^, Mn^2+^, Co^2+^, Ni^2+^ and Zn^2+^, and of these only VO^2+^ was able to catalyze the oxidation of ascorbic acid. Further it is not clear if A^.−^ is an intermediate of the oxidation of ascorbic acid^28–30^ or if ascorbic acid is directly oxidized to DHA^30^. The disproportionation reaction for A^**.**−^ is now well established (R12 and 13, Table 1) and is sufficiently rapid to not be rate limiting in this mechanism. This difference can have a moderate effect on the fitted rate constants; for iron of more consequence is the amount of OH^.^ that is produced.

The stoichiometry for the metal ion-catalysed oxidation reactions of ascorbic acid by oxygen is also debated. Khan and Martell^31^ measured iron and copper-catalyzed oxidation of ascorbic acid by oxygen at pH 2–5.5 and reported the reaction was first-order in ascorbic acid, metal and oxygen. Jameson and Blackburn^28^ investigated the copper-catalyzed oxidation of ascorbic acid by oxygen in 0.1 M potassium nitrate at pH 2–3.5 and found a first-order dependence on copper and ascorbate (AH^−^) and half-order on oxygen. The same rate law (for AH^−^) was investigated by Shtamm et al.^30^ at pH 2.7–4. Consistent with the rate law derived by Jameson and Blackburn^28^ the rate observed by Shtamm et al.^30^ was inversely related to the pH, indicating the rate law only applies to ascorbate. Moreover, Jameson and Blackburn^29^ suggest that the stoichiometry can change depending on the nature and concentration of electrolytes, finding evidence that high concentrations of chloride ions (0.1 M) shifted the dependence from 1st order on AH^−^ to half order on total ascorbic acid (AH_2_ + AH^−^).

#### Transition metal rate constants

Measured and estimated reaction rate constants for AH_2_ and AH^−^ with Fe(III) and Cu(II) in the literature are summarized in Table 3. The literature is divided into values for catalytic reactions, including cases for which it is possible to re-calculate a value given for the redox reaction as a catalytic reaction, and values for the redox reactions. The various catalytic reaction stoichiometries as well as pH dependencies and other caveats are also shown in Table 3.

### Reactions of the radical anion

The main product of the AH_2_ and AH^−^ reactions is the radical anion, A^**.**−^ (Fig. 1), which disproportionates to form AH^−^ and DHA or AH_2_ and DHA at low pH: $2{\text{A}}^{{ \cdot - }} \mathop \Leftrightarrow \limits^{{H^{ + } }} {\text{AH}}_{2} /{\text{AH}}^{ - } + {\text{DHA}}$ (R12 and R13, Table 1). There is a wide range of values for the equilibrium constant K_12_ in the literature; Foerster et al.^35^ found pH dependent values of 1.6–7.9 × 10^14^ M^-1^ at pH 4–6.4 (recalculated for the form of the equilibrium constant above) and Buettner and Schafer^22^ reported 5 × 10^14^ M^-1^ at pH 7.4. More recently Van der Zee and Van den Broek^36^ found a value of 1.7 × 10^16^ M^-1^ at pH 7.4 using ESR to monitor A^.−^ and improved calibration techniques. We adopt the value from Van der Zee and Van den Broek^36^ for K_12_ and calculate the equilibrium constant for R13 using ${\text{K}}_{13}^{ - 1} = {\text{ K}}_{12}^{ - 1} {\text{K}}_{{{\text{AH}}_{2} }}$, where ${\text{K}}_{{{\text{AH}}_{2} }}$ is the first dissociation constant of ascorbic acid. We include both reactions in the model due to the highly pH-dependent A^.−^ decay rates. For the forward reactions $2{\text{A}}^{ \cdot - } + {\text{H}}^{ + } \to {\text{AH}}^{ - } + {\text{DHA}}$ (R12) and $2{\text{A}}^{ \cdot - } + 2{\text{H}}^{ + } \to {\text{AH}}_{2} + {\text{DHA}}$ (R13), we use 7 × 10^4^ and 8 × 10^7^ M^−1^ s^−1^ based on the radiolysis study by Bielski et al.^37^. The rate constants are chosen from the two plateaus in the pH dependent rate constants they derived^37^.

### Other model uncertainties

For the model presented here, some of the chemistry is well established, including much of the ROS chemistry, acid–base equilibria, inorganic iron chemistry, and probe and buffer chemistry. There are several general sources of error and uncertainty for the set of reactions in Tables 1 and 2, in addition to the specific uncertainties described above. These include errors in the rate constants, which range from a few percent to a factor of ten or more. In some cases, reaction stoichiometries and product distributions are also uncertain. Measurement data was often collected in solutions containing other solutes that may impact reaction rates and/or reaction mechanisms, but how and at what concentrations other solutes effect the rates is not known. Temperature differences also introduce uncertainties when data are collected at different temperatures. Further, usually only a small number of the uncertain reactions are important for a given set of experimental conditions.

**Table 2:** Ascorbic Acid Model (continued).

#	Reaction	$k_{f}$	$k_{r}$	$K_{eq}$
**ROS reactions**				
23i	${\text{OH}}^{ \cdot } + {\text{OH}}^{ \cdot } = {\text{H}}_{2} {\text{O}}_{2}$	5.5E9		
24j	${\text{H}}_{2} {\text{O}}_{2} + {\text{OH}}^{ \cdot } = {\text{ HO}}_{2}^{ \cdot } + {\text{H}}_{2} {\text{O }}$	3.2E7		
25i	${\text{O}}_{2}^{ \cdot - } + {\text{OH}}^{ \cdot } = {\text{OH}}^{ - } + {\text{O}}_{2}$	1.01E10		
26i	${\text{HO}}_{2}^{ \cdot } + {\text{OH}}^{ \cdot } = {\text{H}}_{2} {\text{O}} + {\text{O}}_{2}$	7.1E9		
27i	${\text{O}}_{2}^{ \cdot - } + {\text{H}}_{2} {\text{O}}_{2} = {\text{OH}}^{ - } + {\text{OH}}^{ \cdot } + {\text{O}}_{2}$	0.13		
28k	${\text{O}}_{2}^{ \cdot - } + {\text{O}}_{2}^{ \cdot - } = {\text{O}}_{2} + {\text{H}}_{2} {\text{O}}_{2} - 2{\text{H}}^{ + }$	6.0E5		
29i	${\text{HO}}_{2}^{ \cdot } + {\text{O}}_{2}^{ \cdot - } = {\text{HO}}_{2}^{ - } + {\text{O}}_{2}$	9.7E7		
30l	${\text{H}}_{2} {\text{O}}_{2} + {\text{ HO}}_{2}^{ \cdot } = {\text{H}}_{2} {\text{O}} + {\text{O}}_{2} + {\text{OH}}^{ \cdot }$	0.5		
31i	${\text{HO}}_{2}^{.} + {\text{HO}}_{2}^{.} = {\text{O}}_{2} + {\text{H}}_{2} {\text{O}}_{2}$	8.3E5		
32i	${\text{O}}_{2}^{2 - } + {\text{H}}^{ + } = {\text{HO}}_{2}^{ - }$	1E10		
33m	${\text{HSO}}_{4}^{ - } + {\text{OH}}^{ \cdot } = {\text{ SO}}_{4}^{ .- } + {\text{H}}_{2} {\text{O}}$	3.5E5		
**General equilibria**				
34m	${\text{H}}_{2} {\text{O}} \rightleftharpoons {\text{H}}^{ + } + {\text{OH}}^{ - }$	1.3E−3	1.3E11	1E−14
35l	${\text{H}}_{2} {\text{O}}_{2} \rightleftharpoons {\text{H}}^{ + } + {\text{HO}}_{2}^{ - }$	1.26E−2	1E10	1.26E−12
36j	${\text{HO}}_{2} \rightleftharpoons {\text{H}}^{ + } + {\text{O}}_{2}^{ \cdot - }$	1.14E6	7.2E10	1.58E−5
37l	${\text{H}}^{ + } + {\text{SO}}_{4}^{2 - } \rightleftharpoons {\text{HSO}}_{4}^{ - }$			9.77E1
**Inorganic Fe(II)/Fe(III) reactions**				
38m	${\text{Fe}}^{3 + } + {\text{H}}_{2} {\text{O}} \rightleftharpoons {\text{FeOH}}^{2 + } + {\text{H}}^{ + }$			6.11E−3
39m	${\text{FeOH}}^{2 + } + {\text{H}}_{2} {\text{O}} \rightleftharpoons {\text{Fe}}\left( {{\text{OH}}} \right)_{2}^{ + } + {\text{H}}^{ + }$			7.78E−6
40l	${\text{Fe}}^{2 + } + {\text{H}}_{2} {\text{O}} \rightleftharpoons {\text{ FeOH}}^{ + } + {\text{H}}^{ + }$			3.16E−10
41l	${\text{Fe}}^{3 + } + {\text{SO}}_{4}^{2 - } \rightleftharpoons {\text{FeSO}}_{4}^{ + }$			8.32E3
42l	${\text{Fe}}^{3 + } + 2{\text{SO}}_{4}^{2 - } \rightleftharpoons {\text{Fe}}({\text{SO}}_{4} )_{2}^{ - }$			2.63E5
43l	${\text{Fe}}^{2 + } + {\text{SO}}_{4}^{2 - } \rightleftharpoons {\text{FeSO}}_{4}$			1.78E2
44m	${\text{Cl}}^{ - } + {\text{Fe}}^{3 + } \rightleftharpoons {\text{FeCl}}^{2 + }$	3E3	2.16E3	1.39
45n,k	${\text{Fe}}^{2 + } + {\text{O}}_{2} = {\text{Fe}}^{3 + } + {\text{O}}_{2}^{ \cdot - }$	1E−4 (1 < pH < 4, 37 °C) n		
		3.9 (pH 7.0, 37 °C) k,n		
46l	${\text{Fe}}\left( {{\text{III}}} \right)^{*} + {\text{O}}_{2}^{ \cdot - } = {\text{Fe}}^{2 + } + {\text{O}}_{2}$	5E7		
47l	${\text{FeSO}}_{4}^{ + } + {\text{O}}_{2}^{ \cdot - } = {\text{Fe}}^{2 + } + {\text{SO}}_{4}^{2 - } + {\text{O}}_{2}$	< 1E3^∆^		
48l	${\text{Fe}}({\text{SO}}_{4} )_{2}^{ - } + {\text{O}}_{2}^{ \cdot - } = {\text{Fe}}^{2 + } + 2 {\text{SO}}_{4}^{2 - } + {\text{O}}_{2}$	< 1E3^∆^		
49l	${\text{Fe}}\left( {{\text{III}}} \right)^{*} + {\text{HO}}_{2}^{ \cdot } = {\text{Fe}}^{2 + } + {\text{O}}_{2} + {\text{H}}^{ + }$	2E4		
50l	${\text{FeSO}}_{4}^{ + } + {\text{HO}}_{2}^{.} = {\text{Fe}}^{2 + } + {\text{SO}}_{4}^{2 - } + {\text{O}}_{2} + {\text{H}}^{ + }$	< 1E3^∆^		
51l	${\text{Fe}}({\text{SO}}_{4} )_{2}^{ - } + {\text{HO}}_{2}^{.} = {\text{Fe}}^{2 + } + 2{\text{SO}}_{4}^{2 - } + {\text{O}}_{2} + {\text{H}}^{ + }$	< 1E3^∆^		
52l	${\text{Fe}}^{3 + } + {\text{H}}_{2} {\text{O}}_{2} \rightleftharpoons {\text{Fe}}\left( {{\text{HO}}_{2} } \right)^{2 + } + {\text{H}}^{ + }$	3.1E7	1E10	3.1E−3
53l	${\text{FeOH}}^{2 + } + {\text{H}}_{2} {\text{O}}_{2} \rightleftharpoons {\text{Fe}}\left( {{\text{OH}}} \right)\left( {{\text{HO}}_{2} } \right)^{ + } + {\text{H}}^{ + }$	2E6	1E10	2E−4
54l	${\text{Fe}}\left( {{\text{II}}} \right)^{*} + {\text{OH}}^{ \cdot } = {\text{ Fe}}^{3 + } + {\text{OH}}^{ - }$	2.7E8		
55l	${\text{FeSO}}_{4} + {\text{OH}}^{ \cdot } = {\text{ Fe}}^{3 + } + {\text{ SO}}_{4}^{2 - } + {\text{OH}}^{ - }$	2.7E8		
56l	${\text{Fe}}\left( {{\text{II}}} \right)^{*} + {\text{O}}_{2}^{ \cdot - } = {\text{Fe}}^{3 + } + {\text{O}}_{2}^{2 - }$	1E7		
57l	${\text{FeSO}}_{4} + {\text{O}}_{2}^{ \cdot - } = {\text{Fe}}^{3 + } + {\text{SO}}_{4}^{2 - } + {\text{O}}_{2}^{2 - }$	5E8		
58l	${\text{Fe}}\left( {{\text{II}}} \right)^{*} + {\text{HO}}_{2}^{.} = {\text{ Fe}}^{3 + } + {\text{HO}}_{2}^{ - }$	1.2E6		
59l	${\text{FeSO}}_{4} + {\text{HO}}_{2}^{.} = {\text{ Fe}}^{3 + } + {\text{SO}}_{4}^{2 - } + {\text{HO}}_{2}^{ - }$	1.2E6		
60l	${\text{Fe}}^{2 + } + {\text{H}}_{2} {\text{O}}_{2} = {\text{Fe}}^{3 + } + {\text{OH}}^{ \cdot } + {\text{OH}}^{ - }$	55		
61	${\text{FeOH}}^{ + } + {\text{H}}_{2} {\text{O}}_{2} = {\text{Fe}}^{3 + } + {\text{OH}}^{ \cdot } + 2{\text{OH}}^{ - }$	55 (same as R60)		
62l	${\text{FeSO}}_{4} + {\text{H}}_{2} {\text{O}}_{2} = {\text{Fe}}^{3 + } + {\text{ SO}}_{4}^{2 - } + {\text{OH}}^{ \cdot } + {\text{OH}}^{ - }$	78		
63l	${\text{Fe}}\left( {{\text{HO}}_{2} } \right)^{2 + } = {\text{HO}}_{2}^{.} + {\text{Fe}}^{2 + }$	2.3E−3		
64l	${\text{Fe}}\left( {{\text{OH}}} \right)\left( {{\text{HO}}_{2} } \right)^{ + } = {\text{Fe}}^{2 + } + {\text{HO}}_{2}^{.} + {\text{OH}}^{ - }$	2.3E−3		
**Copper chemistry**				
65o	${\text{Cu}}^{2 + } + {\text{H}}_{2} {\text{O}} \rightleftharpoons {\text{CuOH}}^{ + } + {\text{H}}^{ + }$			1.12E−8
66o	${\text{Cu}}^{2 + } + 2{\text{H}}_{2} {\text{O}} \rightleftharpoons {\text{Cu}}\left( {{\text{OH}}} \right)_{2} + 2{\text{H}}^{ + }$			6.31E−17
67o	${\text{Cu}}^{2 + } + 3{\text{H}}_{2} {\text{O}} \rightleftharpoons {\text{Cu}}\left( {{\text{OH}}} \right)_{3}^{ - } + 3{\text{H}}^{ + }$			2.51E−27
68o	${\text{Cu}}^{2 + } + 4{\text{H}}_{2} {\text{O}} \rightleftharpoons {\text{Cu}}\left( {{\text{OH}}} \right)_{4}^{2 - } + 4{\text{H}}^{ + }$			1.82E−40
69o	$2{\text{Cu}}^{2 + } + {\text{H}}_{2} {\text{O}} \rightleftharpoons {\text{Cu}}_{2} {\text{OH}}^{3 + } + {\text{H}}^{ + }$			3.98E−7
70o	$2{\text{Cu}}^{2 + } + 2{\text{H}}_{2} {\text{O}} \rightleftharpoons {\text{Cu}}_{2} \left( {{\text{OH}}} \right)_{2}^{2 + } + 2{\text{H}}^{ + }$			3.72E−11
71o	$3{\text{Cu}}^{2 + } + 4{\text{H}}_{2} {\text{O}} \rightleftharpoons {\text{Cu}}_{3} \left( {{\text{OH}}} \right)_{4}^{2 + } + 4{\text{H}}^{ + }$			7.94E−22
72o	${\text{Cu}}^{2 + } + {\text{SO}}_{4}^{2 - } \rightleftharpoons {\text{CuSO}}_{4}$			223.9
73o	${\text{Cu}}^{2 + } + {\text{Cl}}^{ - } \rightleftharpoons {\text{CuCl}}^{ + }$			6.76
74o	${\text{Cu}}^{2 + } + 2{\text{Cl}}^{ - } \rightleftharpoons {\text{CuCl}}_{2}$			3.98
75p	${\text{Cu}}\left( {{\text{II}}} \right)^{*} + {\text{OH}}^{ \cdot } \rightleftharpoons {\text{CuOH}}^{2 + }$	3.5E8	3E4	1.17E4
76p	${\text{Cu}}\left( {{\text{II}}} \right)^{*} + {\text{HO}}_{2}^{.} = {\text{Cu}}^{ + } + {\text{O}}_{2} + {\text{H}}^{ + }$	1E8		
77q,r	${\text{Cu}}\left( {{\text{II}}} \right)^{*} + {\text{H}}_{2} {\text{O}}_{2} = {\text{Cu}}^{ + } + {\text{O}}_{2}^{ \cdot - } + 2{\text{H}}^{ + }$	< 1^∆^ (q,r) for ${\text{Cu}}^{2 + }$, ${\text{CuOH}}^{ + }$ and ${\text{CuSO}}_{4}$ 70 (q) for ${\text{CuCl}}^{ + }$ and ${\text{CuCl}}_{2}$		
78p	${\text{Cu}}^{ + } + {\text{O}}_{2} \rightleftharpoons {\text{Cu}}^{2 + } + {\text{O}}_{2}^{ \cdot - }$	4.6E5	8E9	
79p	${\text{Cu}}^{ + } + {\text{OH}}^{ \cdot } = {\text{Cu}}^{2 + } + {\text{OH}}^{ - }$	3E9		
80s	${\text{Cu}}^{ + } + {\text{H}}_{2} {\text{O}}_{2} = {\text{Cu}}^{2 + } + {\text{OH}}^{ \cdot } + {\text{OH}}^{ - }$	< 100^∆^		
81t	${\text{Cu}}^{ + } + {\text{H}}_{2} {\text{O}}_{2} = {\text{Cu}}^{3 + } + 2{\text{OH}}^{ - }$	61		
82t	${\text{Cu}}^{ + } + {\text{Cu}}^{3 + } = 2{\text{Cu}}^{2 + }$	3.5E9		
83m	${\text{Cu}}^{ + } + {\text{HO}}_{2}^{.} = {\text{Cu}}^{2 + } + {\text{H}}_{2} {\text{O}}_{2} - {\text{H}}^{ + }$	2.3E9		
84p	${\text{Cu}}^{ + } + {\text{O}}_{2}^{ \cdot - } = {\text{Cu}}^{2 + } + {\text{H}}_{2} {\text{O}}_{2} - 2{\text{H}}^{ + }$	9.4E9		
**Other reactions specific to a subset of experiments**				
85u	${\text{HEPES}} \rightleftharpoons {\text{H}}^{ + } + {\text{HEPES}}^{ - }$			1E−3
86u	${\text{HEPES}}^{ - } \rightleftharpoons {\text{H}}^{ + } + {\text{HEPES}}^{2 - }$			2.73E−8
87v	${\text{BA}} \rightleftharpoons {\text{H}}^{ + } + {\text{BA}}^{ - }$			6.3E−5
88v	${\text{BA}} + {\text{OH}}^{ \cdot } = {\text{products}}$	1.03E9		
89v	${\text{BA}}^{ - } + {\text{OH}}^{ \cdot } = {\text{products}}$	4.66E9		
90w	${\text{H}}_{3} {\text{PO}}_{4} \rightleftharpoons {\text{H}}^{ + } + {\text{H}}_{2} {\text{PO}}_{4}^{ - }$			7.08E−3
91w	${\text{H}}_{2} {\text{PO}}_{4}^{ - } \rightleftharpoons {\text{H}}^{ + } + {\text{HPO}}_{4}^{2 - }$			6.31E−8
92w	${\text{HPO}}_{4}^{2 - } \rightleftharpoons {\text{H}}^{ + } + {\text{PO}}_{4}^{3 - }$			4.79E−13
93x	${\text{H}}_{2} {\text{PO}}_{4}^{ - } + {\text{OH}}^{ \cdot } = {\text{H}}_{2} {\text{PO}}_{4}^{ \cdot } + {\text{OH}}^{ - }$	2E4		
94x	${\text{HPO}}_{4}^{2 - } + {\text{OH}}^{ \cdot } = {\text{HPO}}_{4}^{ \cdot - } + {\text{OH}}^{ - }$	1.5E5		
95y	${\text{PO}}_{4}^{3 - } + {\text{OH}}^{ \cdot } = {\text{PO}}_{4}^{ \cdot 2 - } + {\text{OH}}^{ - }$	7E6		

Fe(II)* represents ${\text{Fe}}^{2 + }$ and ${\text{FeOH}}^{ + }$, Fe(III)* represents ${\text{Fe}}^{3 + }$, ${\text{FeOH}}^{2 + }$, ${\text{Fe}}\left( {{\text{OH}}} \right)_{2}^{ + }$ and ${\text{FeCl}}^{2 + }$.

${\text{Cu}}\left( {{\text{II}}} \right)^{*}$ represents ${\text{Cu}}^{2 + }$, ${\text{CuOH}}^{ + }$, ${\text{CuSO}}_{4}$, CuCl^+^ and CuCl_2_.

These forward and back reactions are written separately in the KPP input file.

^∆^An upper limit is used for these reactions.

^i^Gonzalez et al.^40^,^j^Miller et al.^58^,^k^Pham and Waite^51^,^l^De Laat and Le^38^,^m^Herrmann et al.^43^,^n^Stumm and Morgan^50^,^o^Powell et al.^42^,^p^Deguillaume et al.^41^,^q^Wang et al.^59^,^r^Lee et al.^60^,^s^Pham et al.^61^,^t^Pham et al.^62^,^u^Goldberg et al.^63^,^v^Wu et al.^64^,^w^Skogareva et al.^65^,^x^Morozov and Ershov^66^,^y^Kochany and Lipczynska-Kochany^67^.

Some of our validation data was collected at 37 °C to mimic physiological conditions. Unfortunately, almost all literature data available for the set of reactions used here were reported for room temperature, and temperature dependencies were not available. Because temperature dependence is reaction specific, and can even have different signs, we have only adjusted the small number of rate coefficients for which there is temperature dependence. Gas solubility is also temperature dependent; we use a dissolved oxygen content corresponding to the temperature of each experiment.

## Methods

### Model description and extraction of rate coefficients

The model (Tables 1 and 2) includes reactions that describe the chemistry of reactive oxygen species, iron, ascorbate, sulfate and chloride, and a few reactions specific to the detection of DHA or OH^⋅^ corresponding to the experimental datasets used to validate the model. The model builds on previous models describing aqueous OH^⋅^ production kinetics in the presence of iron and sulfate^38–40^. Additional reactions describing the copper^41, 42^ and chlorine^43^ chemistry have also been updated. The ascorbic acid mechanism (Fig. 1) is built into the model based on the detailed review of the available literature (described above). The final form of the ascorbic acid-metal reaction is similar to Scheme A; however, we have also explored many other forms and stoichiometries of the reactants (below).

The chemical kinetics mechanisms were solved using the Kinetics Pre-Processor (KPP) 2.2.3^26^ with the gfortran compiler and the Rosenbrock solver. For measurements using two reaction coils in series with different conditions (below), the model was run separately for each set of conditions, and the output of the first segment was used as an input for the second.

To solve for the rate constants of two unknown reactions, such as the two reactions needed for copper (R16 and R17) we employ a two-dimensional binary search algorithm, specifically, we vary the rate constants for the two key reactions on an 11 × 11 grid field. For each grid (a combination of two rate constants), we run the model for each Fe(III) or Cu(II) concentration for which we have a measurement and calculate a mean squared error. The MSE of each unit square is obtained by averaging the MSEs of the nearest four grid points. After one cycle, we arrive at a unit square centered by a minimum MSE and we then divide this square into a new 11 × 11 grid field. The range containing the minimum MSE is narrowed as this process is repeated, and after 4 times the rate constant combination with minimum MSE is determined to be the best fit.

For cases where we need to fit more than two rate constants, the grid search method is not efficient enough, thus, we use a coordinate search method instead. We start from randomly chosen initial rate constants for these reactions, along with an initial search range. Each time we vary one rate constant within the search range while keeping the other rate constants fixed, calculate corresponding MSEs, and update the rate constant with the one that produces minimum MSE. This process was applied to each reaction in turn and when this sees no improvement, we reduce the search range in order to continuously decrease the MSE. Finally, when the search range exceeds our required precision, the optimization stops.

### Validation data

We measured the oxidation of ascorbic acid by Fe(II), (III) and Cu(II) by quantifying the oxidation product dehydroascorbic acid (DHA), as described in detail in Campbell et al.^17^. Briefly, DHA is reacted with *o*-phenylenediamine (oPDA) to produce a highly fluorescent product 3-(1,2-dihydroxyethyl)-fluoro[3,4-b]quinoxaline-1-one (DFQ) with unit yield. DFQ is then quantified via fluorescence spectroscopy.

OH^**⋅**^ formation from ascorbate reactions with Fe(II) and Cu(II) at around pH 7 were reported by Charrier and Anastasio^15^ (2.8 µM OH^**⋅**^ from 1 µM Fe(II) and 14 µM from 1 µM Cu at 24 h) and Lin and Yu^27^ (0.3 µM OH^**⋅**^ from 1 µM Fe(II) at 3.8 h and 8.8 µM from 0.3 µM Cu at 6.3 h); Lin and Yu^27^ also reported ascorbate consumption. While the measurements are difficult to compare due to measurement differences and potential non-linear dependencies on both concentration and reaction time, the results appear to be in good agreement for Fe(II) and weak agreement for Cu(II).

#### Reagents and chemical preparation

All chemicals were obtained from Sigma-Aldrich. Ascorbic acid (≥ 99.0%), Chelex 100 sodium form, 0.1 M HCl solution, 0.1 M NaOH solution, CuSO_4_ (≥ 99.0%), FeSO_4_ (≥ 99%), Fe_2_(SO_4_)_3_ (≥ 98%), *o*-phenylenediamine (≥ 99.5%), DHA (≥ 96%), HEPES (≥ 99.5%) were used as received.

A 200 µM solution of ascorbic acid was prepared in Chelex-resin treated MilliQ water (resistivity ≥ 18.2 MΩ cm^−1^), to ensure as low as possible trace metal concentrations and minimize background DHA formation. The average background concentration of DHA observed in MilliQ water was 2.8 ± 0.8 µM. The ascorbic acid working solution was then adjusted to pH 2.8 or pH 7 with HCl or HEPES buffer, respectively. Ascorbic acid, Fe and Cu solutions were made fresh daily to minimize background DHA levels or to avoid formation of precipitates. oPDA solutions at 46 mM for pH 2.8 and 20 mM for pH 7 were prepared in 0.1 M HCl and were prepared fresh daily. All solutions were prepared in sterilized plastic bottles which were washed with 0.1 M HCl and MilliQ water.

#### Online measurements of Fe(II), Fe(III) and Cu(II)

Measurements of ascorbic acid oxidation by iron and copper were conducted in an online instrument described in detail in Campbell et al.^17^. Briefly, a flow of 1.1 mL/min of 200 µM ascorbic acid is added to an equivalent 1.1 mL/min flow of either Cu(II)SO_4_, Fe(II)SO_4_ or Fe(III)_2_(SO_4_)_3_. The reaction mixture was then incubated in reaction coil-1 (Supplementary Table S2) housed in ethylene glycol for 20 min at 37 °C. After passing through the reaction coil, a solution containing 46 mM oPDA in 0.1 M HCl was added at 1.1 mL/min and mixed with the ascorbic acid/metal reaction mixture for 10 min (at pH 2.8) at room temperature in reaction coil-2 (Supplementary Table S2). DHA formed by the oxidation of ascorbic acid/ascorbate reacted rapidly with oPDA to form the highly fluorescent compound DFQ. The reaction mixture containing DFQ then passes through a fluorescence detection cell (details in Campbell et al.^17^). The extent of ascorbic acid oxidation is then expressed in terms of µM DHA using a DHA calibration curve^17^. A summary of reaction conditions and dilution ratios are presented in Supplementary Table S2.

While water typically contains low levels of H_2_O_2_ (not measured here but generally below 10 nM^44^) in the absence of transition metals, there are no pathways to form radicals either from O_2_ or H_2_O_2_. Consistent with this, in the absence of added metals, DHA formation in the reaction coils was below detection limits.

## Results and discussion

### DHA loss pathways

Observations of the stability of DHA indicate it decreases with increasing pH; in pH 2–4, aqueous DHA solutions are stable for days, while at neutral pH, the half-life of DHA is around 20 min^45, 46^. Our model includes three degradation pathways of DHA: reactions with the hydroxyl radical, H_2_O_2_ and hydrolysis to produce 2,3-diketogulonic acid. For the reaction with hydroxyl radical, we estimate a rate constant of 1 × 10^10^ M^−1^ s^−1^, the diffusion limit. The hydrolysis rate constant is thought to be negligible at low pH but reaches (5.3–5.8) × 10^–4^ s^−1^ at neutral pH^46, 47^. DHA also reacts with H_2_O_2_ with an estimated rate constant of 4.2 × 10^–2^ M^−1^ s^−1^ and oxalyl l-threonate, cyclic oxalyl L-threonate  and oxalate as the main products^45^. Calculated DHA degradation from these three pathways is negligible at low pH. At neutral pH, DHA degradation reaches 29–39% of total DHA formation in the first reaction coil for copper and 28% for iron, with hydrolysis as the main pathway. Although the reaction of DHA and oPDA is thought to be fast, with a reaction time of about 14 s, in the first (20 min) reaction coil where oPDA is absent, DHA degradation is quite significant.

### Ascorbate oxidation via the catalytic, redox or OH^⋅^/HO_2_^⋅^/O_2_^⋅−^ pathways

Several lines of evidence point toward the catalytic reaction, AH_2_/AH^−^ + M^n+^  + O_2_ → DHA + M^n+^  + H_2_O_2_ (-H^+^) R14–17) instead of the redox reaction, AH_2_/AH^−^ + M^n+^ → A⋅^−^ + M^(n−1)+^ + (2) H^+^. Both pathways produce DHA directly, or A^**.**−^ which rapidly disproportionates making DHA, thus the two pathways can often produce similar results. However, the catalytic reaction consumes O_2_ and produces ROS (H_2_O_2_), while the redox reaction provides a pathway to produce the reduced form of the metal.

The evidence in favor of the catalytic reaction for iron is as follows. First, the redox pathway fails to produce enough DHA especially at pH 2.8. This is both because the redox reaction converts Fe(III) to Fe(II), and the system can only slowly reoxidize the Fe(II), so Fe(III) can consume only a limited amount of ascorbic acid. Also, the redox reaction seems to produce A^⋅−^, which only produces DHA with 50% efficiency (R12, 13); for the catalytic reaction production of A^⋅−^ vs. direct formation of DHA is less of a settled question. Second, both the catalytic and redox reactions make more cumulative OH^⋅^ from the Fe(III)—ascorbic acid reactions than observed by Lin and Yu^27^ and Charrier and Anastasio^15^ (discussed more in “Comparison with OH^**∙**^ formation data”), but the catalytic reaction is reasonably close to the observations (“Comparison with OH^**∙**^ formation data”) while the redox reaction vastly overshoots. The catalytic reaction directly generates the OH^**⋅**^ precursor H_2_O_2_, but it does not produce the Fe(II) needed for the Fenton reaction to convert H_2_O_2_ to OH^**⋅**^ as does the redox reaction.

The evidence in favor of the catalytic reaction for copper is as follows. In experiments where the extent of the reaction is high, as for Cu(II) at pH 7 (Fig. 3), DHA formation eventually stops increasing. The catalytic reaction is able to reproduce the general asymptotic shape of the DHA formation dependence on Cu(II) (Fig. 3) while the redox mechanism predicts a linear relationship (this is also observed for Fe(II), but the iron phenomenon likely depends more on autoxidation of Fe(II) to Fe(III) rather than the Fe(III) reacting with ascorbate). The reason the catalytic reaction produces an asymptotic behavior is that oxygen is consumed in the closed reaction tubes, limiting reactions 14–17 (Table 1). Additionally, when we include both the redox and catalytic pathways in the model using the coordinate search algorithm for pH 2.8 Fe(III) and pH 7 Cu(II), the optimization steps in the direction where rate constants for the redox reactions of AH_2_ + Fe(III) and AH^−^ + Cu(II) continuously decrease; for the other DHA data the model cannot differentiate between the catalytic and redox reactions. OH^**⋅**^ production from the copper via both pathways falls between the two divergent observational results^15, 27^. Further support for the catalytic mechanism comes from Jameson and Blackburn^28^, who reported that although some degree of charge transfer occurred within the copper-ascorbate complex, the complete one-electron redox reaction did not happen when there was no oxygen.

ROS reactions are only important in the experimental systems if OH^**⋅**^, HO_2_^.^ and/or O_2_^**⋅**−^ concentrations are high, such as for conditions associated with experiments with added H_2_O_2_, or when Fe(II) is the dominant form of iron, for example. Were more DHA formation to be attributed to ROS chemistry, much higher concentrations of OH^**⋅**^ would be needed, a situation clearly not supported by the OH^**⋅**^ measurements by both Charrier and Anastasio^15^ and Lin and Yu^27^. Our result indicates that for both Fe(III) and Cu(II), the catalytic pathway is always dominant compared to the ROS pathway, by 4–6 and 1–3 orders of magnitude for Fe(III) and Cu(II) respectively. However, in the Fe(II) case, the contribution of the catalytic pathway and ROS pathways are more comparable; the ratio of catalytic to ROS pathways decreases from 11 at 2.5 µM Fe(II) to 1.4 at 200 µM Fe(II). This is because Fe(II) produces ROS via reduction of molecular oxygen and the Fenton reaction, pathways not available to Fe(III).

While the oxidized form of the Fe and Cu might be expected to dominate ascorbate consumption in many situations, the contributions of OH^**⋅**^, HO_2_^**⋅**^ and/or O_2_^**⋅**−^ may be significant under some conditions. Given the rate constants (Table 1), OH^**⋅**^, HO_2_^**⋅**^/O_2_^**⋅**−^ need to be ~ 10^–9^, 10^–4^ × [Fe(III)] or 10^–8^, 10^–3^ × [Cu(II)], respectively to account for around half of ascorbate loss. OH^**⋅**^, HO_2_^**⋅**^ in liquid phases in equilibrium with gas phase concentrations of 10^6^ and 10^7^ molec/cm^3^ result in liquid phase concentrations of ~ 10^–3^ and 10^–2^ nM, respectively^48^. However, even with modest concentrations of organics, the radicals will be rapidly depleted away from the interface^48^. Consistent with this, model calculations for lung lining fluid estimated concentrations of 10^–10^–10^–7^ nM for OH^**⋅**^, 10^–6^–10^–4^ nM for HO_2_^**⋅**^ and 10^–3^–10^–1^ nM for O_2_^**⋅**−^, with the highest concentrations associated with extremely high PM concentrations^33^. In comparison a typical concentration for iron and copper is around several micromolar in the bronchoalveolar lavage and can be even higher when exposed to highly polluted environments, and thus should usually be the dominant sink for ascorbic acid and ascorbate^49^.

### Catalytic reaction rate constants

#### Fe(III)

**Figure 2:**
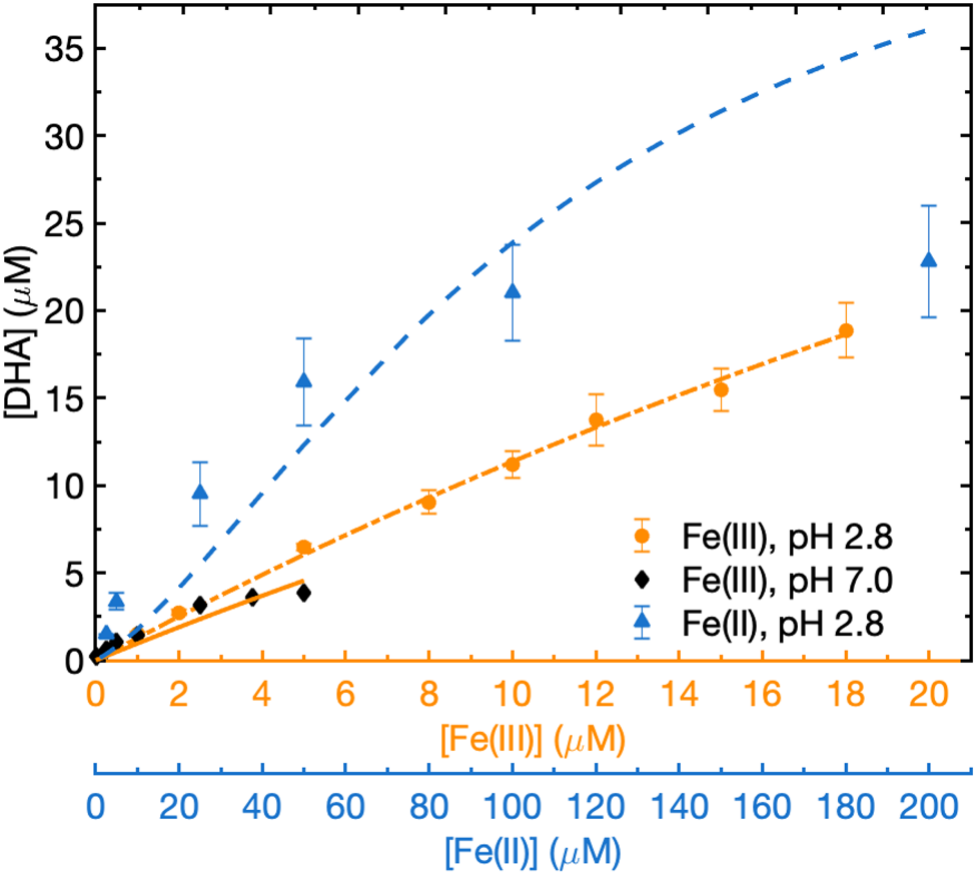
DHA formation from ascorbic acid oxidation in the presence of Fe(III) or Fe(II) ions. The circles, diamonds and triangles represent experimental data for Fe(III) at pH 2.8 and 7.0 (orange abscissa), Fe(II) at pH 2.8 (blue abscissa), respectively. Error bars are shown for data with three measurements; measurements at pH 7 are only one repeat. The orange dashed line, orange solid line and blue dashed line denote corresponding modeling results based on reactions in Tables 1 and 2. Because of the additional uncertainties and lack of relevance of the high Fe(II) concentrations, only experimental data for Fe(II) concentration within 200 µM at pH 2.8 is shown in the figure.

In the Fe(III)/AH_2_ system, the model is very sensitive to the catalytic reactions AH_2_ (R14) or AH^−^ (R15) or both, depending on pH. We minimize the sum of MSEs for the two iron data sets (Fe(III) at pH 2.8 and 7) to derive rate constants for the catalytic reactions R14 and R15. Best-fit third-order rate constants [Fe(III)] [AH_2_/AH^−^][O_2_] for R14, R15 are 5.7 × 10^4^ and 4.7 × 10^4^ M^−2^ s^−1^, respectively; these values produce good agreement with the DHA formation data for both Fe(III) and the Fe(II) over the concentration range as shown in Fig. 2. The AH^−^ result is in good agreement (within 35%) with 3.5 × 10^4^ M^−2^ s^−1^ from Buettner^32^, assuming a first order oxygen dependence in their study (Table 3). Our values for AH_2_ and AH^−^ are significantly lower than 4.0 × 10^5^ and 2.4 × 10^7^ M^−2^ s^−1^ reported by Khan and Martell^31^ and the AH^-^ value after recalculation for the catalytic pathway from Lakey et al.^33^ of 2.3 × 10^5^ M^−2^ s^−1^, Table 3.

#### Fe(II) + O_2_

Because the asymptotic behavior observed for very high Fe(II) at pH 2.8 is far outside the relevant range for environmental samples, we include only Fe(II) data up to 200 µM. The model is reasonably successful at reproducing DHA formation from Fe(II) at pH 2.8 at lower Fe(II) concentrations (Fig. 2), although it overpredicts DHA formation at high Fe(II) (> 100 µM). Ascorbate is only oxidized by OH^**⋅**^, HO_2_^**⋅**^, O_2_^−^ and Fe(III), so in the presence of Fe(II) ascorbate oxidation should be controlled by the production of Fe(III) or ROS. Both of these pathways are initiated by the reduction of O_2_ via R45 and enhanced by H_2_O_2_ production from Fe(III) + ascorbic acid (R14, 15). The rate constant for R45, Fe(II) + O_2_ is pH sensitive. Stumm and Morgan^50^ suggest that when pH > 5 the rate increases with pH, with a second-order dependence on OH^−^ concentration. At low pH (1–4), this rate constant is ~ 10^–5^ M^−1^ s^−1^ and independent of pH. This rate constant has been found to increase by a factor of 10 for a 15 °C temperature increase^50^. Therefore, for pH 2.8 we use a rate constant of 10^–5^ M^−1^ s^−1^ at room temperature and 10^–4^ M^−1^ s^−1^ at 37 °C. For pH 7.0, a k_45_ of 0.39 M^−1^ s^−1^ was suggested by Pham and Waite^51^ which we adjusted upward to 3.9 M^−1^ s^−1^ at 37 °C.

The Fe(II) measurements (Fig. 2) show that initial AH_2_ consumption/DHA production by Fe(II) is around one sixth of that of Fe(III), however, this likely results partly from conversion of Fe(II) to Fe(III) in the stock solution. Although the Fe(II) stock solution was made fresh daily, in the few hours required for the measurements Fe(II) slowly oxidizes to Fe(III). Rate constants for R45 at room temperature are ~ 0.11 M^−1^ s^−1^ for pH 6.0 and 0.17 M^−1^ s^−1^ for pH 6.5^51^. The pH of our 1 mM FeSO_4_ stock solution is ~ 6.3, so about 13%/h of Fe(II) is oxidized to Fe(III). Because the degree of oxidation of the stock solution was not the same for each experiment, this may explain the larger error bars for Fe(II) (Fig. 2). The modelling results for Fe(II) shown here are based on the assumption that 10% of Fe(II) was oxidized to Fe(III) before each experiment.

#### Copper catalytic reaction rate constants

Compared to iron, there are more studies of the copper reactions with ascorbic acid, and more disagreements (Table 3). Here, the rate laws from Jameson and Blackburn^28^, Jameson and Blackburn^29^, Shtamm et al.^30^ and Khan and Martell^31^ are all tested. For simplicity, we use overall reactions for the catalytic pathway; the products of the catalytic reaction are H_2_O_2_ and either DHA or A^**∙**−^, depending on the charge balance of the equations.

**Figure 3:**
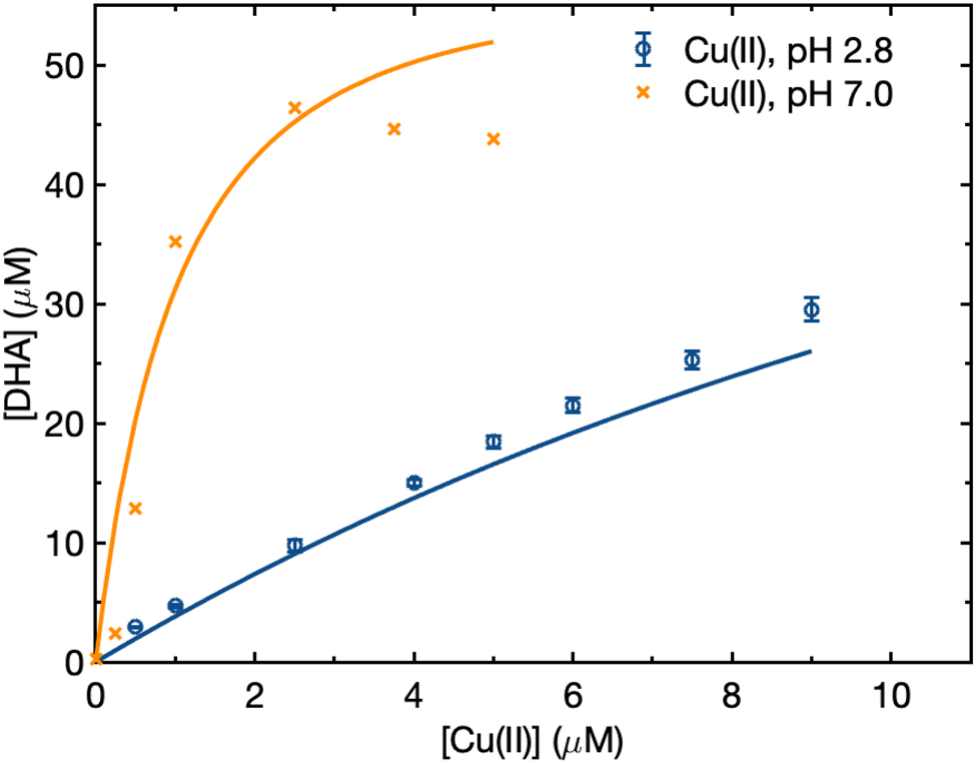
DHA formation from ascorbic acid in the presence of Cu(II). The circles and crosses represent experimental data for Cu(II) at pH 2.8 and Cu(II) at pH 7.0, respectively, and lines indicate corresponding model results based on the reactions shown in Tables 1 and 2. Error bars are shown for data with three measurements; measurements at pH 7 are only one repeat. The abscissa represents Cu(II) concentration in the first reaction coil and ordinate represents DHA formation in the second reaction coil.

Ascorbic acid oxidation by Cu(II) is more efficient than by Fe(III) (Figs. 2 and 3). For the 1:1:1 stoichiometry first proposed by Khan and Martell^31^, the third-order rate constants [Cu(II)][AH_2_/AH^−^][O_2_] for R16 and R17 that provide the best fit of the data are 7.7 × 10^4^ and 2.8 × 10^6^ M^−2^ s^−1^, respectively (Fig. 3). The Cu(II) ion catalyzes the oxidation of ascorbic acid more efficiently at neutral pH than acidic pH; this is reflected in the much higher value for k_17_ than k_16_. In the pH 7.0 measurements, the theoretical maximum DHA concentration in the second reaction coil is 100 μM, but similar to the observation for Fe(II), DHA formation reached a much lower maximum of about 45 μM at 2.5 µM Cu(II). The model matches this behavior well for Cu(II), due to a combination of the depletion of oxygen in the solution which suppresses DHA production at high Cu(II) concentrations, and the hydrolysis of DHA, which is significant at high pH.

**Table 3:** Summary of rate constants for reactions of iron and copper with ascorbic acid.

	Fe(III) + AH_2_	Fe(III) + AH^−^	Cu(II) + AH_2_		Cu(II) + AH^−^		Notes
**Catalytic Reaction: (AH**_**2**_ **or AH**^**−**^**)**^**x**^**+ (Fe(III) or Cu(II)) + **(O _2_)^y^** → (DHA)**^**x** ^**+ (Fe(III) or Cu(II)) **+ (H_2_**O**_2_)^**z**^							
Khan and Martell^31^	AH_2_/AH^−^ + Fe(III)/Cu(II) + O_2_ → DHA + Fe(III)/Cu(II) + H_2_O_2_ (-H^+^)						
	4.0 × 10^5^ M^−2^ s^−1^	2.4 × 10^7^ M^−2^ s^−1^	3.8 × 10^5^ M^−2^ s^−1^		6.0 × 10^7^ M^−2^ s^−1^		
Jameson and Blackburn^28^	AH^−^ + Cu(II) + $\frac{1}{2}$ O_2_				4.3 × 10^3^ M^−3/2^ s^−1^		0.1 M nitrate
Jameson and Blackburn^29^	$\frac{1}{2}$[AH^−^]_TOT_ + Cu(II) + $\frac{1}{2}$ O_2_		4.5 M^−1^ s^−1^, pH 1.81 to 63 M^−1^ s^−1^, pH 3.85				0.1 M chloride
Shtamm et al.^30^	AH^−^ + Cu(II) + $\frac{1}{2}$ O_2_				5.2 × 10^3^ M^−3/2^ s^−1^		
Buettner^32^*		3.5 × 10^4^ M^−2^ s^−1^			3.1 × 10^6^ M^−2^ s^−1^		k recalc. w/1st order dependence on O_2_
Lakey et al.^33^*		2.3 × 10^5^ M^−2^ s^−1^			3.0 × 10^6^ M^−2^ s^−1^		k recalc. w/1st order dependence on O_2_
**Redox Reaction AH**_**2**_**/AH**^**−**^ **+ Fe(III)/Cu(II) →A**^**⋅−**^ **+ Fe(II)/Cu(I) + (2) H**^**+**^ **M**^**−1**^ **s**^**−1**^							
Buettner^32^		10			880		pH 7 solution with O_2_
Sun et al.^68^		4.5 × 10^3^					@ pH 4, but AH_2_ or AH^−^ not specified
Lakey et al.^33^		66			8.4 × 10^2^		mix of Fe(III)/Fe(IV)

*The reaction appears to be catalytic, but these papers assumed a redox reaction; the rate has been recalculated here assuming an oxygen dependence.

The fitted rate constants for AH_2_ and AH^−^ are lower than 3.8 × 10^5^ and 6.0 × 10^7^ M^−2^ s^−1^ in Khan and Martell^31^. Using a first order oxygen dependence, (redox) rate constants for Cu(II) and AH^−^ in Buettner^32^ and Lakey et al.^33^ can be converted into (catalytic) third-order rate constants k([Cu(II)][AH^−^][O_2_]) of 3.1 × 10^6^ and 3.0 × 10^6^ M^−2^ s^−1^ respectively, in good agreement with the rate constant we derived. Both the iron and copper data suggest our experimental data are in better agreement with measurement of Buettner^32^, while there might be an overestimation of the rate constants in Khan and Martell^31^.

If we instead use the rate law [Cu(II)][AH^−^][O_2_]^1/2^ suggested by Jameson and Blackburn^28^ and Shtamm et al.^30^ with a 2.5 order rate constant we find a best-fit value for AH^−^ of 1.2 × 10^5^ M^−3/2^ s^−1^. This value is about 1.5 orders of magnitude larger than 4.3 × 10^3^ M^−3/2^ s^−1^ from Jameson and Blackburn^28^ and 5.2 × 10^3^ M^−3/2^ s^−1^ from Shtamm et al.^30^. Some or all of the difference could be due to the difference in temperature; as the earlier measurements were made at ~ 25 °C and ours were mostly at 37 °C or from the low concentrations of chloride ions^5^ included in our experiments.

Nevertheless, this 1:1:0.5 rate law predicts higher DHA formation at high Cu(II) than observed in the pH 7.0 data. This is partly because the reaction’s half-order oxygen dependence means O_2_ is less depleted and is less able to limit DHA formation. The 1:1:1 stoichiometry of Cu(II), AH_2_/AH^−^ and oxygen fits the shape of DHA formation curve better and arrives at a smaller MSE. However, the room temperature DHA hydrolysis rate used in the model may also underestimate DHA consumption.

Jameson and Blackburn^29^ suggested a [Cu(II)][AH_2_ + AH^−^]^1/2^[O_2_]^1/2^ (1:0.5:0.5) rate law for solutions containing 0.1 M chloride ions. Our experiment includes chloride ions as follows: for pH 2.8 experiments the pH in the first reaction coil was adjusted with HCl resulting in 1.6 × 10^–3^ M Cl^−^, and the oPDA mixed into the 2nd reaction coil is prepared in 0.1 M HCl increasing the Cl^−^ concentration in the 2nd coil by 0.03 and 0.05 M for pH 2.8 and 7 experiments respectively, but DHA forms in the first reaction coil, so this is less important. This stoichiometry provides a much worse fit for our data than the other rate laws, implying our concentrations of Cl^−^ are too low to significantly alter the Cu(II)-ascorbic acid reaction.

### Comparison with OH^⋅^ formation data

To further test the reactions we derived with data from the literature, we include benzoate (the OH^**⋅**^ probe) and phosphate buffer reactions (R87–95) and calculate OH^**⋅**^ production for the conditions used by Charrier and Anastasio^15^ and Lin and Yu^27^. Although both of these studies only investigated Fe(II), our model includes reactions that oxidize this species to Fe(III), and thus we can use the data to constrain the products of the Fe(III) + AH_2_/AH^−^ reaction. With A^**⋅**−^ + HO_2_^**⋅**^ as products, the model produces OH^**⋅**^ that exceeds the observations from both Charrier and Anastasio^15^ and Lin and Yu^27^ by 2 orders of magnitude, suggesting the products are H_2_O_2_ + DHA. The H_2_O_2_ + DHA combination of products produces 4 µM OH^**⋅**^ formation from 1 µM Fe(II) after 24 h, in reasonable agreement with the Charrier and Anastasio^15^ measurement of 2.8 µM. A similar calculation for the Lin and Yu^27^ conditions overshoots the reported OH, resulting in a concentration that is about a factor of three higher, and somewhat overestimates consumption of ascorbate (the modeled concentration at 3.8 h is about 90% of the measured value). The discrepancy may be due to several factors, including the temperature difference, errors in various rate constants or the observational data, or incorrect assumptions about the products of the Fenton reaction; there is some evidence in the literature that the Fenton reaction can produce either OH^**⋅**^ or Fe(IV), with a pH dependent product distribution. Fe(IV) production may be favored around neutral pH^52–54^. Our model only considers OH^**⋅**^ as a product and could thus overestimate its formation.

For copper, the model results fall between the OH^**⋅**^ formation measurements from Charrier and Anastasio^15^ and Lin and Yu^27^, which are widely divergent from each other, at about 2.6 times the former and 27% of the latter. Although both the Lin and Yu^27^ data and our model agree at nearly 100% ascorbic acid loss at ~ 6 h, ascorbic acid concentration predicted by the model decreases with time nonlinearly, different from the linear trend in the experiment. Several other aspects of Cu(I) and Cu(II)—ROS chemistry are uncertain; some additional discussion of the gaps can be found in the SI.

## Implications and conclusions

In recent years, there has been widespread application of acellular assays to measure particle-bound ROS and aerosol oxidative potential (OP), with measurements spanning large spatial, temporal and chemical spaces^11, 18, 55^. OP is proving to be better at predicting adverse health impacts than particle mass^11, 18^. The complex interplay between OP assays, including the ascorbic acid assay, and redox-active PM components has widely been demonstrated^14, 18, 56^. Detailed understanding of assay responses is crucial to fully elucidate both the role of chemical composition on aerosol OP and to directly probe the role of aerosol OP in particle toxicity. Ultimately, this information should be translatable into policy that specifically targets the components in PM that drive OP (and are associated with higher levels of toxicity), but this is only possible if there is a clear understanding of what each OP assay responds to. Thus, a firm mechanistic understanding of the chemistry underlying OP assays is urgently required to deconvolute and interpret the complex reactivity of OP assays. In future, the model described here in conjunction with online measurements of aerosol OP and PM composition in the ambient atmosphere, will provide a detailed understanding of AA oxidation by ambient PM.

## Supplementary Information

Supplementary Information.

## Data Availability

All data generated or analyzed during this study are included in this published article (and its Supplementary Information files).
