# Supplementary material for: Ascorbate oxidation by iron, copper and reactive oxygen species: review, model development, and derivation of key rate constants
Source: Sci Rep. 2021 Apr 1;11:7417. doi: 10.1038/s41598-021-86477-8 (PMC8016884; doi:10.1038/s41598-021-86477-8)
Supplement: Supplementary file 1 — Supplementary Information. [file 41598_2021_86477_MOESM1_ESM.pdf]

Supplementary information for the manuscript "Ascorbate Oxidation by Iron, Copper and Reactive Oxygen Species: Review, Model Development, and Derivation of Key Rate Constants"

Jiaqi Shen,[1] Paul T. Griffiths,[2] Steven J. Campbell,[3] Battist Utinger,[3] Markus Kalberer[2, 3] and Suzanne E. Paulson[1*]

[1]Department of Atmospheric and Oceanic Sciences, University of California at Los Angeles, Los Angeles, CA USA 90095-1565
[2]Department of Chemistry, Cambridge University, Lensfield Rd. Cambridge CB2 1EW UK
[3]Department of Environmental Sciences, University of Basel, Klingelbergstrasse 27, 4056 Basel, Switzerland

*Corresponding Author. paulson@atmos.ucla.edu; Telephone: (310) 206 4442; fax (310) 206 5219

1. Additional Mechanisms for Cu Reactions with Ascorbic Acid Proposed in the Literature

Jameson and Blackburn[1]

$$\mathrm{Cu^{2+} + AH^- \rightleftharpoons [Cu(AH)]^+} \qquad \text{Eq(7)}$$

$$\mathrm{2[Cu(AH)]^+ \rightleftharpoons [Cu_2(AH)_2]^{2+}} \qquad \text{Eq(8)}$$

$$\mathrm{[Cu_2(AH)_2]^{2+} + O_2 \rightleftharpoons [Cu_2(AH)_2(O_2)]^{2+}} \qquad \text{Eq(9)}$$

$$\mathrm{[Cu_2(AH)_2(O_2)]^{2+} \rightarrow [CuA(O_2H)]^{\cdot} + Cu^{2+} + A^{\cdot -} + H^+} \qquad \text{Eq(10)}$$

$$\mathrm{A^{\cdot -} + [Cu_2(AH)_2(O_2)]^{2+} \rightarrow [CuA(O_2H)]^{\cdot} + [Cu(AH)]^+ + DHA} \qquad \text{Eq(11)}$$

$$\mathrm{[CuA(O_2H)]^{\cdot} \rightarrow A^{\cdot -} + HO_2^- + Cu^{2+}} \qquad \text{Eq(12)}$$

$$\mathrm{2[CuA(O_2H)]^{\cdot} \rightarrow termination\ products} \qquad \text{Eq(13)}$$

$$\mathrm{H^+ + HO_2^- \rightleftharpoons H_2O_2} \qquad \text{Eq(14)}$$

Scheme C Shtamm et al.[2]

$$2Cu^{2+} + AH^- \rightarrow 2Cu^+ + DHA + H^+ \qquad Eq(15)$$

$$Cu^+ + O_2 \rightarrow CuO_2^+ \qquad Eq(16)$$

$$CuO_2^+ + AH^- \rightarrow Cu^+ + DHA + HO_2^- \qquad Eq(17)$$

$$CuO_2^+ + Cu^+ + 2H^+ \rightarrow 2Cu^{2+} + H_2O_2 \qquad Eq(18)$$

2. Fluorescence Measurement

The fluorescent product 3-(1,2-dihydroxyethyl)-fluoro[3,4-b]quinoxaline-1-one (DFQ) is excited at $\lambda = 365$ nm by a high-power UV LED, with a strong peak emission at $\lambda_{max} = 430$ nm measured using optical fibers (Thorlabs M93L01) coupled to a femtowatt photoreceiver (Thorlabs type PDF10A) with a cyan fluorescent protein excitation filter (Thorlabs type MF434-17), which only transmits light between 423 and 445 nm. For pH 7 the mixture containing DFQ is measured with a spectrometer (Ocean Optics QE Pro) with an integration time of 1000 ms and an integration interval from 425 to 435 nm.

3. Copper – ROS Reactions

OH· production from Cu(II) is very sensitive to Cu(II) + H_2O_2 (R77) which produces Cu(I) and the reactions between Cu(I) and H_2O_2 (R80-81) which together control the formation of OH·. Pham et al.[3] found a high rate constant for R77 of 460 $M^{-1}s^{-1}$, but most studies agree that the rate constant for copper and copper hydroxide species is < 1 $M^{-1}s^{-1}$ [4-7] and 70 $M^{-1}s^{-1}$ for the Cu(II)-chloro complex;[5] we adopt these values here (Tab. 2). Similar to the iron Fenton reaction, Cu(III) is also thought to be a possible product of the reaction between Cu(I) and H_2O_2 with a rate constant of 61 $M^{-1}s^{-1}$;[3] only an upper limit rate constant (100 $M^{-1}s^{-1}$) could be found for the reaction of Cu(I) and $H_2O_2 \rightarrow$ OH· (R80[8]). Relatively larger uncertainties exist in the copper Fenton reaction than iron.

Other potential sources of error can be the uncertainties of the rate law associated with the background ions (see manuscript).

Table S1. Rate constant for OH· + AH_2/AH^-

pH	Rate constant (M^{-1} s^{-1})	Reference
1.0	7.9 ×10^{9}*	Redpath and Willson[9]
7.0	10^{9}	
7.4	1.1 ×10^{10}*	Buettner and Schafer[10]
1.0	7.2 ×10^{9}	Bielski[11]
1.5	4.5×10^{9}	Bielski[11]
7.0	7.0 ×10^{9}	
7.0	1.1 ×10^{10}	Bielski[11]
11.0	4.1 ×10^{9}	Bielski[11]
7.4	1.1 ×10^{10}	Lakey et al.[7]

* Rate constants adopted in this study.

Table S2. Reaction conditions

**Final reaction conditions: reaction coil-1**				
	**pH 2.8**		**pH 7**	
Ascorbic acid concentration	100	μM	200	μM
**Flow rates:**				
Ascorbic acid	1.1	ml/min	0.5	ml/min
DHA/Cu(II)/Fe(II)/Fe(III)	1.1	ml/min	0.5	ml/min

**Temp:**	37	°C	37	°C
**Reaction time:**	20	min	20	min
**Final reaction conditions: reaction coil-2**				
Ascorbic acid concentration	67	µM	100	µM
oPDA concentration	15	mM	10	mM
**Flow rates:**				
Ascorbic acid	1.1	ml/min	0.5	ml/min
DHA/Cu(II)/Fe(II)/Fe(III)	1.1	ml/min	0.5	ml/min
oPDA	1.1	ml/min	1	ml/min
**Temp:**	20	°C	37	°C
**Reaction time:**	10	min	5	min

References

1. Jameson, R.F. and Blackburn, N.J., Role of copper dimers and the participation of copper (III) in the copper-catalysed autoxidation of ascorbic acid. Part II. Kinetics and mechanism in 0.100 mol dm–3 potassium nitrate. *Journal of the Chemical Society, Dalton Transactions,* 1976a(6): p. 534-541.
2. Shtamm, E., Purmal, A., and Skurlatov, Y.I., Mechanism of catalytic ascorbic acid oxidation system Cu2+–ascorbic acid–O2. *International Journal of Chemical Kinetics,* 1979. **11**(5): p. 461-494.
3. Pham, A.N., Xing, G., Miller, C.J., and Waite, T.D., Fenton-like copper redox chemistry revisited: hydrogen peroxide and superoxide mediation of copper-catalyzed oxidant production. *Journal of catalysis,* 2013. **301**: p. 54-64.
4. Zhou, S., Yu, Y., Sun, J., Zhu, S., and Deng, J., Oxidation of microcystin-LR by copper (II) coupled with ascorbic acid: Kinetic modeling towards generation of H2O2. *Chemical Engineering Journal,* 2018. **333**: p. 443-450.
5. Wang, Z., *et al.*, Accelerated oxidation of 2, 4, 6-trichlorophenol in Cu (II)/H2O2/Cl- system: A unique “halotolerant” Fenton-like process? *Environment international,* 2019. **132**: p. 105128.
6. Lee, H., *et al.*, Activation of oxygen and hydrogen peroxide by copper (II) coupled with hydroxylamine for oxidation of organic contaminants. *Environmental science & technology,* 2016. **50**(15): p. 8231-8238.
7. Lakey, P.S., *et al.*, Chemical exposure-response relationship between air pollutants and reactive oxygen species in the human respiratory tract. *Scientific reports,* 2016. **6**: p. 32916.
8. Pham, A.N., Rose, A.L., and Waite, T.D., Kinetics of Cu (II) reduction by natural organic matter. *The Journal of Physical Chemistry A,* 2012. **116**(25): p. 6590-6599.
9. Redpath, J. and Willson, R., Reducing compounds in radioprotection and radio-sensitization: Model experiments using ascorbic acid. *International Journal of Radiation Biology and Related Studies in Physics, Chemistry and Medicine,* 1973. **23**(1): p. 51-65.
10. Buettner, G.R. and Schafer, F.Q., Ascorbate (Vitamin C), its antioxidant chemistry. *Society For Free Radical Biology and Medicine,* 2001: p. 20.
11. Bielski, B.H., Chemistry of ascorbic acid radicals. *Adv. Chem. Ser.;(United States),* 1982. **200**.
